# Special Hospital for Officers Suffering from Shock—Rejoinder to Dr. Ash

**Published:** 1914-12-19

**Authors:** 


### Special Hospital for Officers Suffering from Shock.

### Rejoinder to Dr. Ash.

*To the Editor of* The Hospital.

Sir,—Dr. Ash misses altogether the whole point of this controversy. The question is not whether traumatic neurosis and psychosis can be treated successfully in the existing special hospitals for nervous diseases, but whether they can be more ideally treated under conditions which do not obtain at these hospitals. Such patients have doubtless been cured in a nursing home in a noisy street. But surely more ideal conditions are conceivable? Anyhow it is quite enough for me, whatever it may be for Dr. Ash, that the eminent members of his profession who are on the staff of this hospital think that separate accommodation is advisable. As I have already pointed out, one of them is on the staff of Welbeck Street, two are on the staff of the National; another member of that staff and other well-known members of the staff of existing hospitals for nervous diseases have offered their services, which have been gratefully accepted. So on this point I think Dr. Ash is out of court.

Dr. Ash quotes Professor Dejerine as an upholder of the sufficiency of cubicle accommodation. Whatever the medical profession may have done in the past, no one to-day can question the quality of Professor Dejerine's work. I have the honour to know him, and I have spent a morning with him at his hospital, and he kindly showed me all his cases and his method of treating them. It may have been mere accident, but there were none of his cases which were comparable for one moment with men returning from the Front suffering from shock. Perfect and absolute quiet, for instance, prevailed in those long wards of curtained cubicles. But, confidently, I say that no one who has seen Professor Dejerine's wards at that great hospital could hold them, or it, up as worthy of imitation in this country. And every child in hospital work knows that conditions which are apparently successful in foreign cliniques and hospitals are not at all necessarily suitable to our national temperament.

I thank Dr. Ash for telling me of the compliment paid to me by his friends who asked him whether I was a doctor with special experience in nervous diseases. This shows that what I wrote was sense, and showed knowledge—gained, of course, from those behind me. None of my friends have asked the same question about Dr. Ash after reading his first letter.

True, as Dr. Ash writes, there are many eminent neurologists whose names might have been added to the staff. *Hinc illæ lachrymæ?*

"A great point has been made," he writes, "of the fact that even if the existing hospitals can deal with these patients only the neurologists constituting their staffs could carry out the treatment." Who made this "great" point? I did not. What I wrote was that Dr. Ash was wrong in saying that if the patients were admitted into the present hospitals any professional man outside the staff of those hospitals would be allowed to supervise their treatment—a very different matter. The War Office are opening a special hospital at Moss Side, near Liverpool, for the treatment of soldiers suffering from shock, and have asked us to restrict 10 Palace Green to officers—another reason for separate rooms. Dr. Ash asks what are the special treatments that are to be used. He must know perfectly well that with such physicians as are on the staff no treatment will be used which is not recognised by every neurologist and psychologist. All that will be aimed at is that the treatment shall be efficient under ideal conditions, and that there shall be sufficient staff to give individual attention to all the patients.—Yours faithfully,

Knutsford.

[This correspondence must now close.—Ed. The Hospital.]

